# Maternal Vitamin D Deficiency Delays Glomerular Maturity in F1 and F2 Offspring

**DOI:** 10.1371/journal.pone.0041740

**Published:** 2012-08-21

**Authors:** Fernanda A. M. Nascimento, Thais C. Ceciliano, Marcia B. Aguila, Carlos A. Mandarim-de-Lacerda

**Affiliations:** Laboratory of Morphometry, Metabolism and Cardiovascular Disease, Biomedical Center, Institute of Biology, State University of Rio de Janeiro, Rio de Janeiro, RJ, Brazil; Oklahoma State University, United States of America

## Abstract

**Background:**

There is a high prevalence of vitamin D insufficiency in women of reproductive age.

**Methods:**

This work studied the first two generations of offspring (F1 and F2) of Swiss mice from mothers fed one of two diets: SC (standard chow) or VitD- (vitamin D-deficient). Functional and developmental kidney measurements were taken.

**Results:**

The first two generations of the VitD- group had higher blood pressure at 6 months of age than the offspring of the SC group as well as an increase in renin and AT1r expression. However, at all ages, both F1 and F2 VitD- mice had shorter glomerular diameters, and diet played a significant role in the total variation. Both the F1 and F2 generations of the VitD- group had more immature glomeruli than offspring from the SC group. Immature glomeruli begin to disappear at 10 days, but at this age, F1-VitD- mice had more immature and mature glomeruli than F1-SC mice. At 6 months of age, F1-VitD- mice exhibited more glomeruli, while F2-VitD- mice exhibited the same number of glomeruli as F2-SC mice, but fewer glomeruli compared to the F1-VitD group. Both diet and generation account for the total variation in the number of glomeruli. Decreases in urine output and podocin expression and increases in urea and creatinine in the urine were observed in F1 offspring.

**Conclusion:**

These findings demonstrate that maternal vitamin D deficiency accompanies changes in the renal expression of important factors that may retard the maturation of glomeruli by extending the period of nephrogenesis.

## Introduction

Adolescent and adult women of childbearing age in the United States of America have a high prevalence of vitamin D insufficiency [1], [2]. This insufficiency is likely related to diet [3] and is gaining recognition as a public health problem. The prevalence of vitamin D insufficiency is high even in sunny climates, such as Brazil's, principally in adolescents [4].

Vitamin D is critical for the development of the nervous system [5], [6] and immunological functions [7] during fetal development. Vitamin D during the pregnancy is important to both maternal skeletal preservation and fetal skeletal formation. However, new evidence suggests that vitamin D could be vital to normal fetal development and that vitamin D restriction in this period may affect chronic disease susceptibility post-natal life [8]. Furthermore, recent studies have shown that this restriction can generate “genomic imprinting” in the fetus, which is related to the genesis of chronic diseases in adulthood [9]. Moreover, vitamin D is associated with premature births, obesity and renal dysfunction in adulthood, making it more than an essential fat-soluble vitamin responsible for calcium metabolism [9], [10].

Normal development of the kidney is a highly complex process that requires precise cellular proliferation, differentiation and apoptosis [11]. Among the essential regulators of kidney development are components of the renin-angiotensin system (RAS), podocin (a critical component of the glomerular slit diaphragm) and the Wilms' tumor suppressor gene WT1 [12].

Vitamin D restriction during pregnancy and throughout lactation in rats is associated with an increased number of glomeruli and decreased renal corpuscle size among offspring, although the causes and consequences of this abnormal kidney phenotype remain unknown [13]. Therefore, the adverse effects of vitamin D restriction during kidney development need further exploration. This study investigates the effects of maternal vitamin D deficiency on glomerular development in early postnatal life and its effects on renal structure at maturity. This study focuses on the F1 and F2 generations after F0 maternal vitamin D restriction.

## Materials and Methods

Animal protocols were approved by the Animal Ethics Committee of the State University of Rio de Janeiro (Protocol Number CEA/242/2008), and the procedures were conducted in accordance with the guidelines for experimentation with animals (NIH Publication N°. 85-23, revised 1996). Animals were housed at a controlled temperature (21±1°C) and humidity (60±10%), with a 12 h light/dark cycle and free access to food and water.

### Experimental design

Six-week-old, female, virgin Swiss Webster mice (n = 20, obtained from Oswaldo Cruz Institute Foundation, Rio de Janeiro, RJ, Brazil) were allocated to one of two groups for six weeks: SC (standard chow, fed a diet made according to the AIN93G protocol, including 1,000.0 IU/kg of vitamin D3) [14] or VitD- (vitamin D restricted, fed the same AIN93G diet but without vitamin D3). The vitamin D was added separately only to the SC diet, and the vitamin mixture added to both the SC and VitD- diets did not contain vitamin D. Table 1 shows the nutritional components in each diet, including the dietary composition of vitamin D. The recommended minimum dietary requirement of pregnant mice for vitamin D, according to the AIN93G, is 1,000.0 IU/kg of diet. The SC diet contained 400,000 IU of vitamin D3/g or 0.250 g of vitamin D3/kg of the feed mix, which supplied the recommended levels of vitamin D.

**Table 1:** Composition of the diets.

Nutrient (g/kg)	Diets	
	SC	VitD^−^
Corn starch	397.50	397.50
Casein	200.00	200.00
Starch dextrinated	132.00	132.00
Sucrose	100.00	100.00
Soya bean oil	70.00	70.00
Fiber	50.00	50.00
L-cystine	3.00	3.00
Choline	2.50	2.50
Mineral mix	35.00	35.00
Calcium carbonate	357.00	357.00
Vitamin mix	10.00	10.00
Vitamin D3	0.25	0.00

All the nutrients corresponded to the recommendations of AIN93G for rodents. Abbreviations: SC, standard chow; VitD^−^, vitamin D restricted diet.

These mice were the F0 generation. The diets were produced by PragSolucoes (Jau, SP, Brazil). Male mice of the same age (n = 20) were separated and fed only the SC diet. After the six-week period, the mice were mated overnight. After mating, the presence of a vaginal plug was used to diagnose pregnancy. The dams were separated from the males and fed their respective experimental diets until the 10th day of suckling (lactation), when the VitD- diet was switched to SC. This procedure guaranteed that offspring received the experimental diet only during organogenesis. The litter was reduced at birth to six pups per dam (1∶1 male to female ratio) to ensure adequate and standardized nutrition until weaning.

Pups were sexed by measuring the anogenital distance, which is sexually dimorphic in mice (the male's anogenital distance is approximately twice as long as the female's) [15]. F1 females were separated at 21 days from the SC and VitD- groups to produce the F2 generation (n = 10 females per group). From 21 days of age, F1 females were fed standard chow. At 3 months, they were mated with males to give birth to the F2 generation. The F2 offspring were separated and analyzed in the same way as the F1 offspring. The minimum sample size for each analysis was five animals per group.

Body mass (BM) and naso-anal length (NAL) were evaluated at birth, 10 days, weaning, 3 months and 6 months of age. From three to six months, all animals had systolic blood pressure (BP) measured weekly with a non-invasive tail-cuff plethysmography (Letica LE 5100, Panlab, Barcelona, Spain).

### Urine Measurement

Urine samples were taken at 6 months of age, during the week before euthanasia. One mouse per metabolic cage was kept in a system containing six different metabolic cages (n = 6 per group) for 48 h (to acclimatize) and then left for three days to document water intake and take 24 h urine samples for a volume determination. Urine was collected and centrifuged (120 g for 15 min) to remove the solid waste. The 24 h urine output and water intake were adjusted for BM (mL/g BM) and analyzed for urea, creatinine, uric acid and proteinuria using the colorimetric method (Bioclin System II, Quibasa, Belo Horizonte, MG, Brazil).

### Kidney stereology

We studied the F1 and F2 generations of both diet groups. One male mouse per litter was randomly selected to form the groups (the animals from the same litter were never allocated to the same group). We studied kidney stereology at birth, 10 days and 6 months of age in each generation (n = 6 for each group).

Each mouse was decapitated, and the kidneys were rapidly removed and weighed. Briefly, the left kidneys were fixed in freshly prepared fixative (1.27 M formaldehyde in 0.1 M phosphate buffer, pH 7.2) for 48 h at room temperature, longitudinally divided into two halves, embedded face down in Paraplast plus (Sigma-Aldrich, St Louis, MO, USA), serially sectioned at a nominal thickness of 5 µm and stained with hematoxylin and eosin. The total number of glomeruli was evaluated at 6 months of age. In addition, immature and mature glomeruli were estimated at birth and on day 10, when glomerular development is completed. In these age groups, immature glomeruli were identified as those with a comma-shaped body (when the cells of the proximal pole of the vesicles change their shape and develop a crack) or S-shaped body (when there is a second crack in the distal pole of the comma-shaped body). Mature clusters are vascularized and formed by an increase in the cytoplasm of podocytes and invaginations into the center of mesangial area, creating a sphere [11], [16].

The size of the glomeruli was determined with the same method used to estimate their number, the fractionator/dissector method. The fractionator method was used to estimate the number of glomeruli in a slice (starting with a random number for each 10th section). The total number of glomeruli per kidney, adjusted to the entire organ, was estimated from the analyzed fraction of the kidney [17], [18]. The “fraction” of the kidney that we used to count glomeruli considered the size of the glomeruli. As both juxtamedullary and superficial glomeruli should be counted and the diameter of juxtamedullary glomeruli is greater than that of superficial glomeruli, the right solution is to estimate the size of the glomeruli in a random way and determine their mean size. Using image analysis with an LC Evolution camera on an Olympus BX51 microscope running Image-Pro Plus software version 7.01 for Windows (Media Cybernetics, Silver Spring, MD, USA), glomerular size (mean diameter) was estimated as described elsewhere using a minimum of 100 glomeruli per group among those cut equatorially [19]. Then, we observed the series of slices apart from this mean size to avoid overestimation caused by counting the same glomerulus twice.

### Western blotting analysis

After euthanasia, the right kidneys were taken at birth, weaning and 6 months in both generations to determine total proteins. Proteins were extracted in homogenizing buffer with protease inhibitors. The homogenates were centrifuged at 7380 rpm/5000 g for 20 min at 4°C, and the supernatants were collected. Equal quantities of total protein were resuspended in sample buffer containing SDS, heated for 5 min at 100°C and separated by SDS/PAGE. After electrophoresis, the proteins were electroblotted on to PVDF transfer membranes (Hybond-P; Amersham Biosciences). Each membrane was blocked by incubation in 6% (w/v) non-fat dry milk in Tris-buffered saline (20 mM Tris/HCL [pH 7.4] and 500 mM NaCl) containing 0.05% Tween 20 (TBS-T). After blocking, the membranes were incubated with the follow antibodies: a) polyclonal antibody against rabbit anti-human AT1 receptor (AT1r 306; 43 kDa; sc-579; 1∶1000; Santa Cruz Biotechnology); b) monoclonal antibody against mouse renin (A-1; 38–46 kDa; sc-137252; 1∶1000; Santa Cruz Biotechnology); c) polyclonal antibody against rabbit anti-human podocin (H-130; 42 kDa; sc-21009; 1∶1000; Santa Cruz Biotechnology); and d) polyclonal antibody against rabbit anti-human WT1 (C-19; 52 kDa; sc-192; 1∶500; Santa Cruz Biotechnology). The membranes were washed and incubated with anti-mouse (1∶5000 for renin) or anti-rabbit (1∶8000 for podocin and AT1r; 1∶5000 for WT1) secondary antibodies. Protein expression was detected according to the manufacturer's instructions using an ECL (enhanced chemiluminescence) detection system (Amersham Biosciences). Signals were visualized by chemiluminescence, and the digital images were quantitatively analyzed (Image-Pro Plus version 7.01, Media Cybernetics, Silver Spring, MD, USA). The integral absorbance values were measured.

### Data analysis

Data were tested for normal distribution and homogeneity of variance, and the differences between the groups were analyzed by a one-way analysis of variance (ANOVA) and post-hoc test of Tukey, two-way ANOVA (generation *vs.* diet) and unpaired *t*-tests (GraphPad Prism 5.03, GraphPad Software, La Jolla, CA, USA). A *P*-value≤0.05 was considered statistically significant.

## Results

### Dams and pregnancies

No differences were observed in weight gain, litter size, or the number of dead pups in either generation between the mothers in the SC and VitD- groups (data not shown).

### Offspring biometry

#### Body mass (Figures 1 and 2)

**Figure 1:**
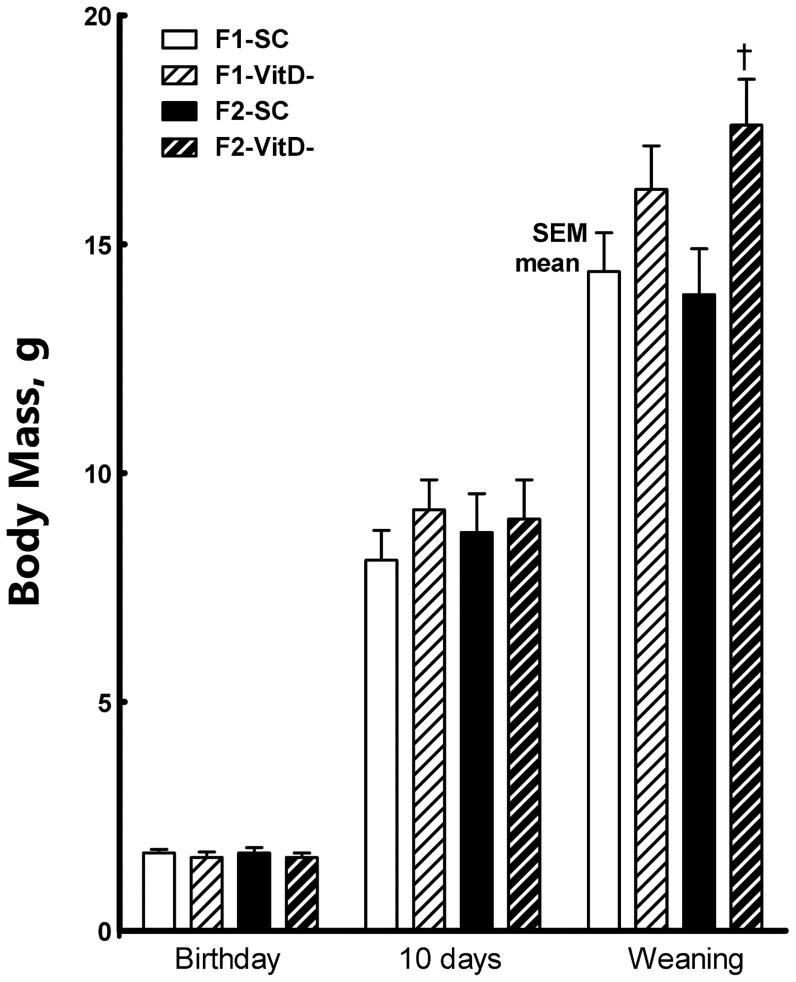
Body mass evolution in both the F1 and F2 generations at earlier stages of development. Data are reported as means ± SEM; P<0.05, one-way ANOVA and post-hoc of Tukey's test: † compared to SC counterpart.

**Figure 2:**
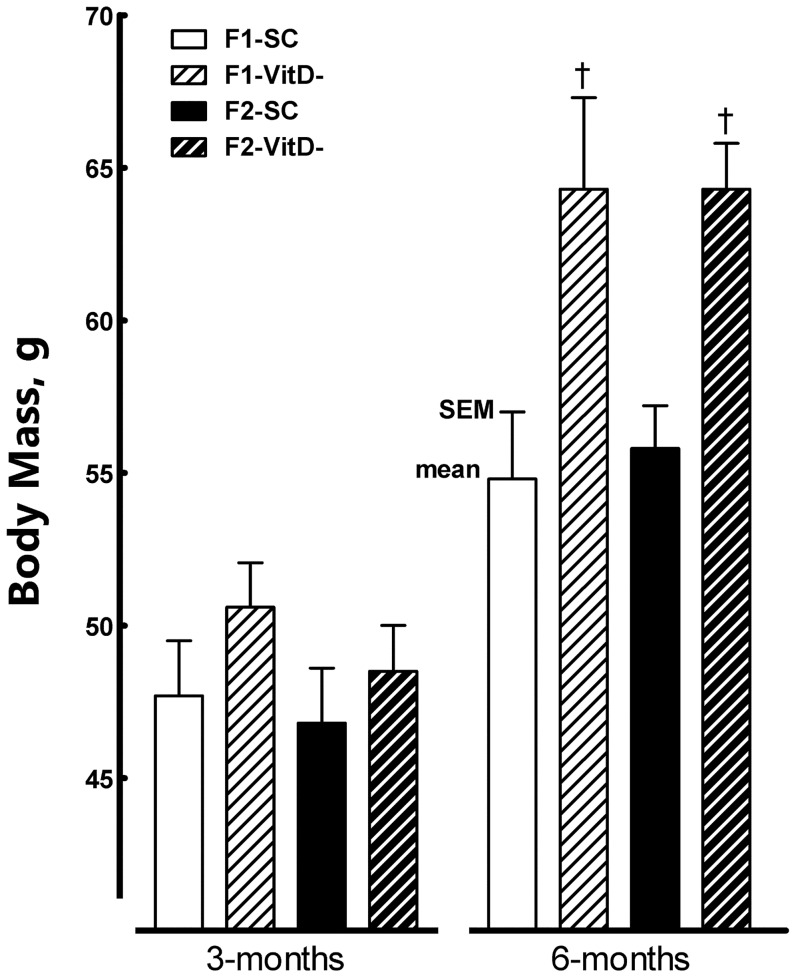
Body mass evolution in both the F1 and F2 generations at 3 and 6 months of age. Data are reported as means ± SEM; P<0.05, one-way ANOVA and post-hoc of Tukey's test: † compared to SC counterpart.

At birth, no differences were observed in either BM or NAL between offspring. There were no differences in NAL throughout the study. At weaning, however, the F2-VitD offspring had higher BMs (+26%) than the F2-SC offspring (*P* = 0.03). At six months, both the F1 and F2 VitD- groups were heavier than their SC counterparts (+17% in F1, +15% in F2, *P*<0.05).

#### Blood Pressure (Figure 3)

**Figure 3:**
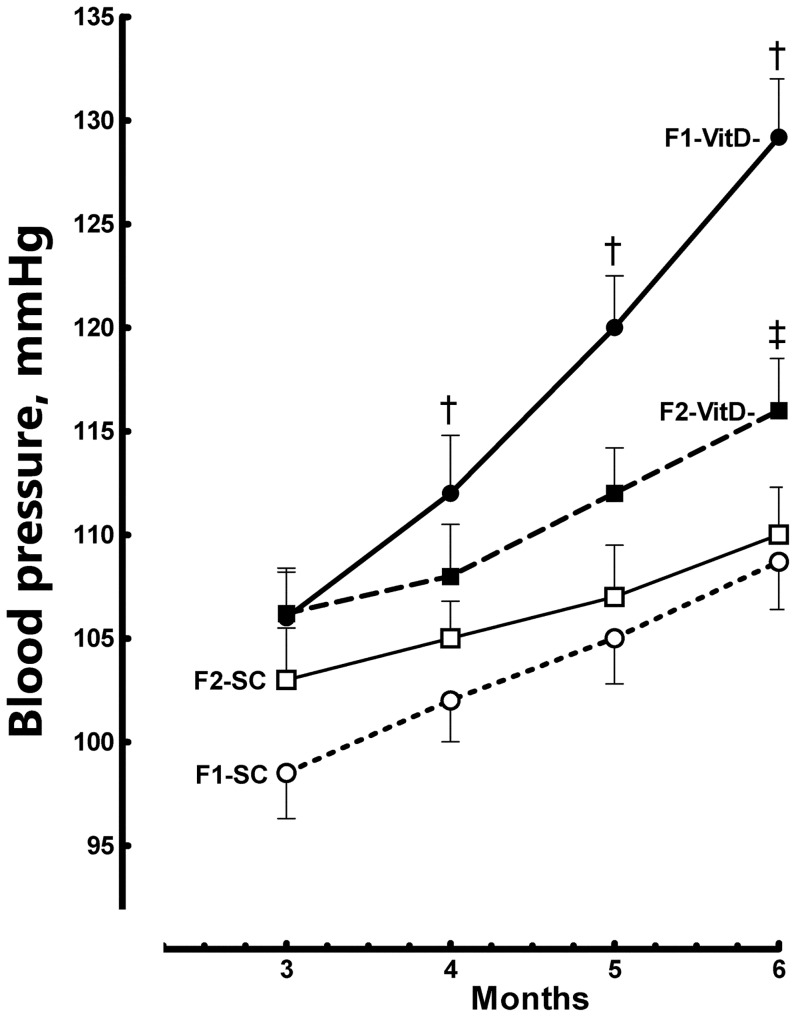
Systolic blood pressure evolution in both the F1 and F2 generations. Data are reported as means ± SEM; P<0.05, one-way ANOVA and post-hoc of Tukey's test: † compared to SC counterpart; ‡ compared to F1 generation.

Blood pressure increased continually from 3 to 6 months of age in both groups. At 4 months, BP was 10% higher in the F1-VitD- group than in the F1-SC group (*P* = 0.04), and this difference continued to increase: at 5 months, it was 15% higher (*P* = 0.001), and at six months, it was 20% higher (*P*<0.0001). The BP of the F2-VitD- offspring increased more slowly than that of the F1-VitD- offspring, and at 6 months, the difference between these groups was significant (F2-VitD- mice had 10% lower BP than F1-VitD- mice, *P*<0.05). The BP of the F2-VitD- mice was significantly higher than that of the F2-SC mice only at 6 months of age (+8%; *P*<0.05).

### Kidney structure

Kidney mass (KM) and glomerular diameter (GD) differed between the VitD- and SC offspring in both the F1 and F2 generations.

#### At birth (Figures 4 and 5)

**Figure 4:**
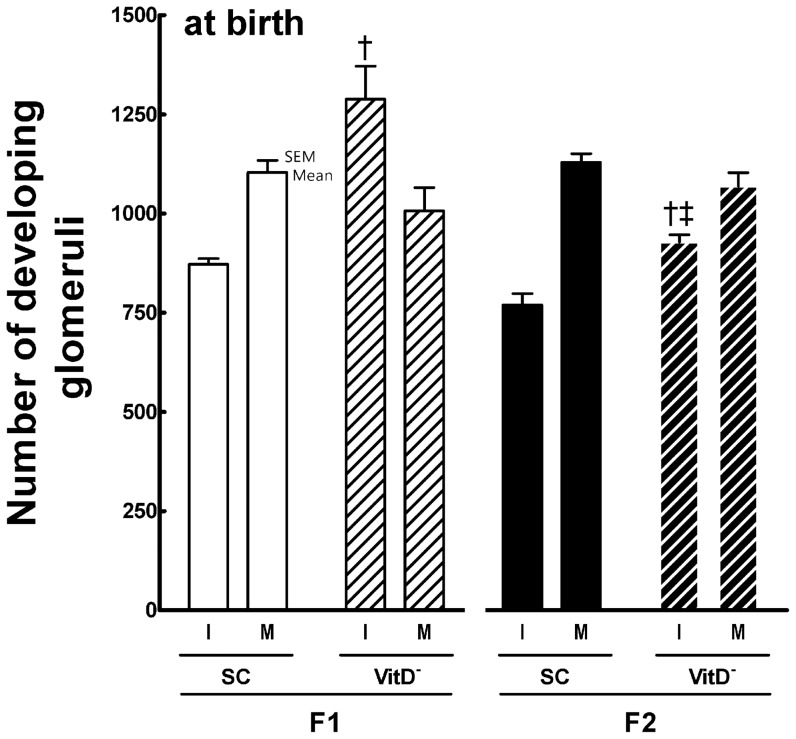
Number of developing immature and mature glomeruli at birth in both the F1 and F2 generations. Data are reported as means ± SEM; P<0.05, one-way ANOVA and post-hoc of Tukey's test: † compared to SC counterpart; ‡ compared to F1 generation.

**Figure 5:**
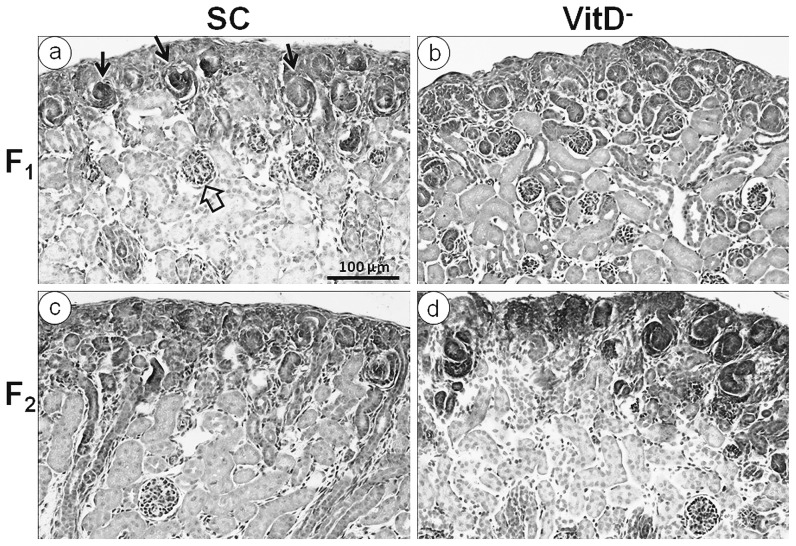
Photomicrographs of the developing kidneys of offspring at birth. Hematoxylin and eosin stain, same magnification in all pictures. (a) SC offspring and (b) VitD- offspring in the F1 generation; (c) SC offspring and (d) VitD- offspring in the F2 generation; (arrows) clusters with comma-shaped and S-shaped glomerular structures at the cortex; (open arrows) vascularized glomerular structures in the inner region.

The mean kidney mass (KM; mean ± SEM, n = 6 animals each group) varied from 8.2±0.2 mg for the F1-SC mice to 10.3±0.4 mg for the F1-VitD- mice (*P*<0.05, *t*-test), with more than 25% variation between the groups (*P*<0.0001). However, no differences were found between the groups in the F2 generation: 8.7±0.4 mg (F2-SC) *vs.* 9.0±0.9 mg (F2-VitD-) (*P*>0.05, t-test). Diet significantly affected KM (*P* = 0.006), but there was no interaction between diet and generation (*P*>0.05; two-way ANOVA).

The mean GD was 12% smaller in the F1-VitD- mice than in the F1-SC mice (43.3±0.3 µm for F1-VitD-mice *vs.* 49.3±0.6 µm for F1-SC mice; *P*<0.05, *t*-test) and 8% smaller in the F2-VitD- mice than in the F2-SC (46.0±0.8 µm and 50.2±1.2 µm, respectively; *P*<0.05, *t*-test). Both diet (*P*<0.0001) and generation (*P* = 0.003) affected the GD, but there was no interaction between diet and generation (*P*>0.05; two-way ANOVA).

There were 50% more immature glomeruli in the F1-VitD- offspring than in the F1-SC offspring (*P*<0.0001, *t*-test) and 20% more immature glomeruli in the F2-VitD- offspring than in the F2-SC offspring (*P*<0.001, *t*-test).

#### At 10 days of age (Figures 6 and 7)

**Figure 6:**
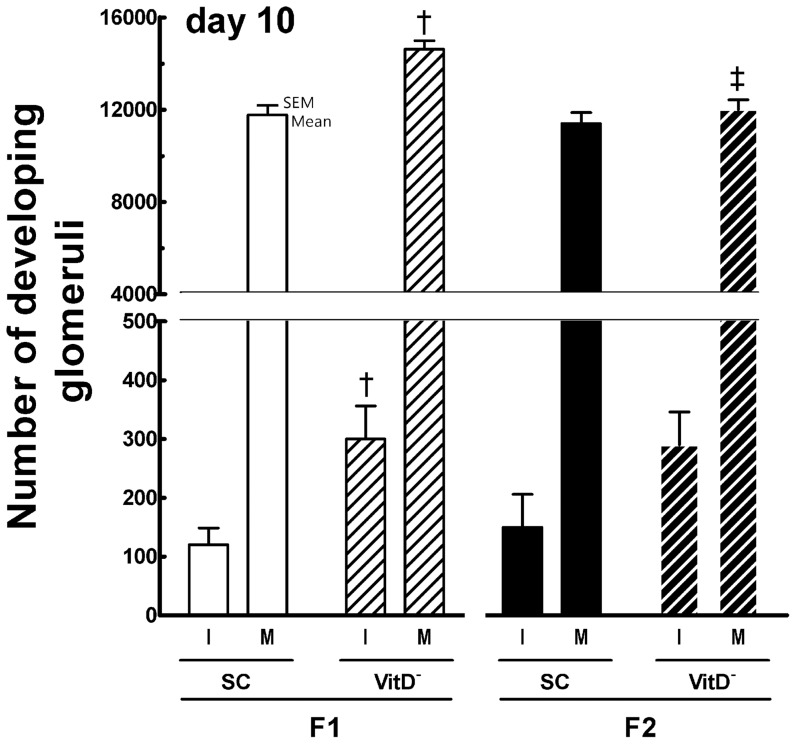
Number of developing immature and mature glomeruli at 10 days in both the F1 and F2 generations. Data are reported as means ± SEM; P<0.05, one-way ANOVA and post-hoc of Tukey's test: † compared to SC counterpart; ‡ compared to F1 generation.

**Figure 7:**
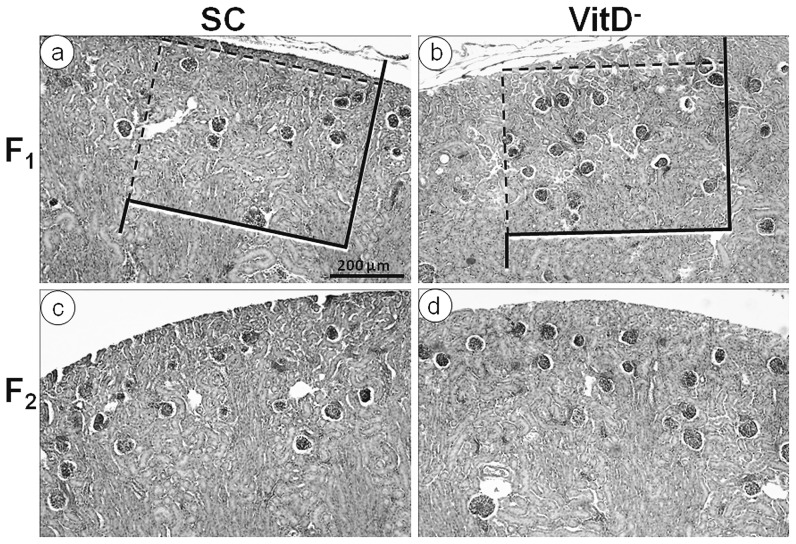
Photomicrographs of developing kidneys from offspring at 10 days of age. Hematoxylin and eosin stain, same magnification in all pictures. (a) SC offspring and (b) VitD- offspring in the F1 generation; (c) SC offspring and (d) VitD- offspring in the F2 generation. We used the same test frame size over the photomicrographs of the two groups to emphasize that the number of glomeruli was greater in the VitD- group than in the SC group. As observed at birth (Fig. 5). Differences between the SC group (c) and the VitD- group (d) are less marked in the F2 generation.

The mean KM (mean ± SEM, n = 6 animals each group) varied from 47.4±2.0 mg for the F1-SC mice to 60.2±1.0 mg for the F1-VitD- mice (more than 27% variation; *P* = 0.009, *t*-test) and from 53.0±2.9 mg for the F2-SC mice to 56.3±3.0 mg for the F2-VitD- mice (*P*>0.05, *t*-test). At 10 days of age, generation contributed significantly to KM (*P*<0.0001), but there was no interaction between diet and generation (*P*>0.05; two-way ANOVA).

The mean GD was 7% smaller (81.0±0.6 µm) in the F1-VitD- mice than in the F1-SC mice (87.2±0.6 µm) (*P*<0.05, *t*-test). The GD also decreased approximately 5% in the F2-VitD- mice (82.8±0.7 µm) in comparison with the F2-SC mice (87.3±1.3 µm) (*P*<0.05, *t*-test). Diet affected the GD in 10-day-old offspring (*P*<0.0001), but there was no interaction between diet and generation (*P*>0.05; two-way ANOVA).

Immature glomeruli normally begin to disappear at this age, but the F1-VitD- mice had 150% more immature glomeruli and 25% more mature glomeruli than the F1-SC mice (*P*<0.0001). The number of mature glomeruli was 20% lower in the F2-VitD- mice than in the F1VitD- mice, and there was no difference in the number of mature glomeruli between the F2-VitD- and F2-SC mice at this age.

#### At 6 months of age

The mean KM (mean ± SEM, n = 6 animals each group) varied from 390.2±27.0 mg for the F1-SC mice to 421.3±21.0 mg for the F1-VitD- mice (*P*>0.05, *t*-test) and from 403.9±29.0 mg for the F2-SC mice to 414.8±29.0 mg for the F2-VitD- mice (*P*>0.05, *t*-test). Diet, generation, and the interaction between diet and generation did not affect the KM significantly at 6 months of age (*P*>0.05; two-way ANOVA).

The GD varied from 102.5±1.9 µm for the F1-SC mice to 93.3±1.4 µm for the F1-VitD- mice (less 9% difference; *P* = 0.03, *t*-test) and from 103.0±1.6 µm for the F2-SC to 98.0±2.2 µm for the F2-VitD- mice (*P*>0.05, *t*-test). Diet significantly affected the GD at 6 months of age (*P* = 0.0008), but there was no interaction between diet and generation (*P*>0.05; two-way ANOVA).

The mean number of glomeruli varied from 10897±614 for the F1-SC mice to 14064±308 for the F1-VitD- mice (30% difference; *P*<0.0001, *t*-test) and from 10466±867 for the F2-SC mice to 12015±785 for the F2-VitD- mice (*P*>0.05, *t*-test). Both diet (*P* = 0.001) and generation (*P* = 0.04) affected the number of glomeruli at 6 months of age, but there was no interaction between diet and generation (*P*>0.05; two-way ANOVA). Moreover, comparing offspring of the same groups at 10 days and 6 months of age, the number of glomeruli was not significantly different (*P*>0.05, one-way ANOVA and post-hoc test of Tukey).

### Urine parameters (Table 2)

**Table 2:** Urine parameters.

Parameters	F1-SC	F1-VitD-	F2-SC	F2-VitD-
**WI, g/100 g BM/day**	16.3±1.0	14.4±1.6	15.2±1.1	13.3±1.3
**Urine output**	0.05±0.002	0.02±0.004†	0.04±0.006	0.04±0.004
**Urea, mg/dL**	36.5±3.8	194.7±23.2†	45.5±8.1	58.2±8.5‡
**Creatinine, mg/dL**	1.2±0.1	3.3±0.2†	1.0±0.1	1.4±0.1‡
**Uric acid, mg/dL**	0.6±0.2	1.2±0.6	0.5±0.1	0.9±0.3
**Proteinuria, g/L**	0.43±0.1	0.55±0.1	0.40±0.1	0.44±0.1

Data were kept for six-month-old male offspring in both the F1 and F2 generations. Data are reported as means ± SEM; *P*<0.05, one-way ANOVA and post-hoc Turkey's test:

† compared to SC counterpart;

‡ compared to F1 generation.

Legends: BM, body mass; FI, food intake; WI, water intake.

In male offspring at six months of age, no differences were found after the 24 h observation of water intake. However, the 24 h urine output, adjusted for BM, was significant lower (−60%; *P* = 0.004) in the F1-VitD- mice than the F1-SC mice, even without differences in water intake between these groups. In addition, the urine urea (+433%, *P*<0.0001) and creatinine (+175%, *P<0.0001*) values were significantly higher in the F1-VitD- mice than in the F1-SC mice. However, the urinary uric acid and proteinuria values were not different across the groups. By the F2 generation, no urinary parameter differed between the VitD- and SC mice. In the F2-VitD- mice, the urine urea (−70%, *P<0.001*) and creatinine (−58%, *P<0.001*) values were decreased in comparison with the F1-VitD- mice.

### Western blotting analyses

The results of protein expression analysis of the F1 and F2 VitD- groups are expressed as a percentage of the result of the F1 and F2 SC counterparts.

All Western blotting data were corrected and normalized to beta actin. Moreover, the beta actin bands are shown in western blotting figures below each protein analyzed. Indeed, beta actin did not differ between the SC and VitD- mice (data not shown).

#### Renin expression (Figure 8) and AT1r expression (Figure 9)

**Figure 8:**
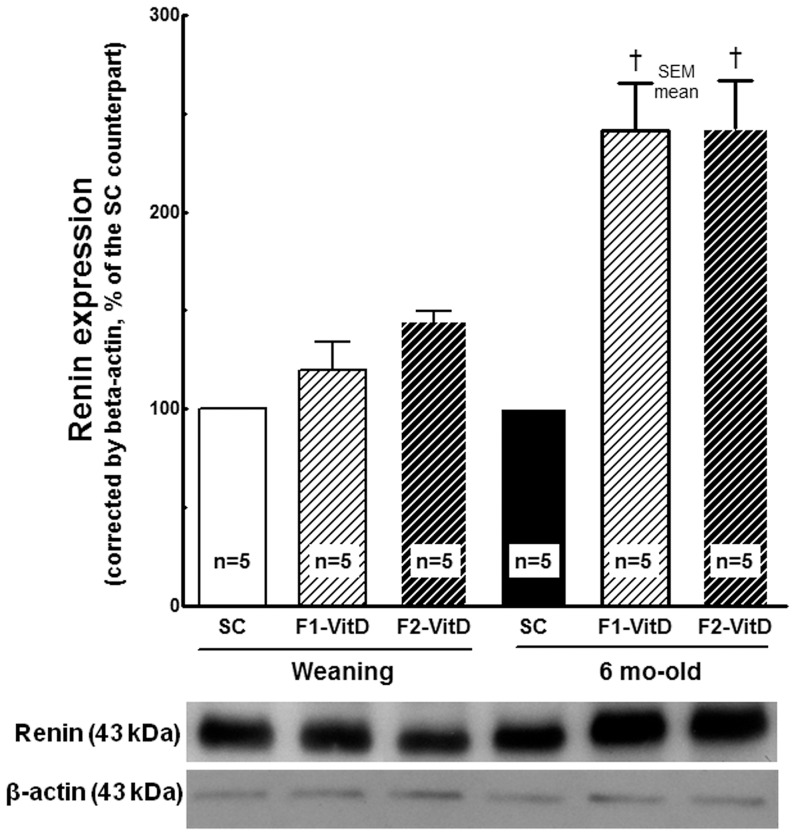
Western blotting analysis of renal tissue at weaning and 6 months of age for renin expression. Average values were measured and equal protein loading was confirmed by probing blots with beta-actin antibody and is expressed as a percentage of the SC counterpart. Data are reported as means ± SEM; P<0.05, one-way ANOVA and post-hoc of Tukey's test: † compared to SC counterpart; ‡ compared to F1 generation.

**Figure 9:**
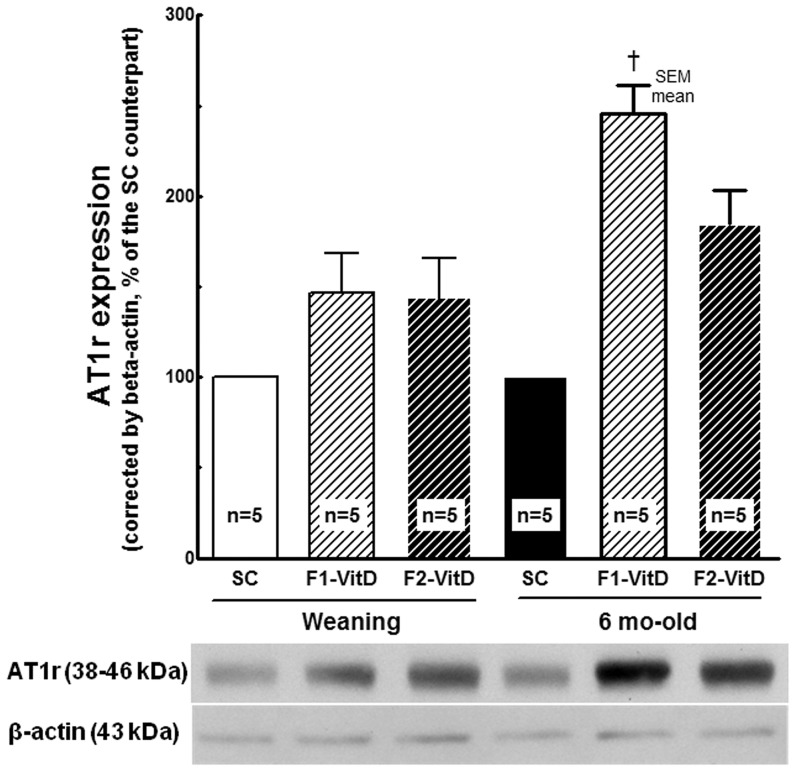
Western blotting analysis of renal tissue at weaning and 6 months of age for AT1 receptor expression. The average values were measured and equal protein loading was confirmed by probing blots with beta-actin antibody and is expressed as a percentage of the SC counterpart. Data are reported as means ± SEM; P<0.05, one-way ANOVA and post-hoc of Tukey's test: † compared to SC counterpart; ‡ compared to F1 generation.

Renin expression was significantly higher at 6 months of age than at weaning in both the F1 and F2 VitD- offspring (*P* = 0.0003, Fig. 8), and this parameter was higher in F1 and F2 VitD- offspring than in the corresponding F1 and F2 SC offspring (*P*<0.001). At weaning, renin expression did not differ between the VitD- and SC mice in either the F1 or F2 generations. However, at 6 month of age, in both the F1 and F2 generations, the VitD- mice had significantly higher values of renin expression than the SC mice (*P* = 0.001).

AT1r expression was significantly higher at 6 months of age than at weaning only in F1-VitD- offspring (*P* = 0.0007, Fig. 9). F1-VitD- offspring displayed higher levels of expression of AT1r than F1-SC offspring (*P*<0.02) There was no difference between F1 and F2 VitD- offspring or F1 and F2 SC offspring.

#### Podocin expression (Figure 10)

**Figure 10:**
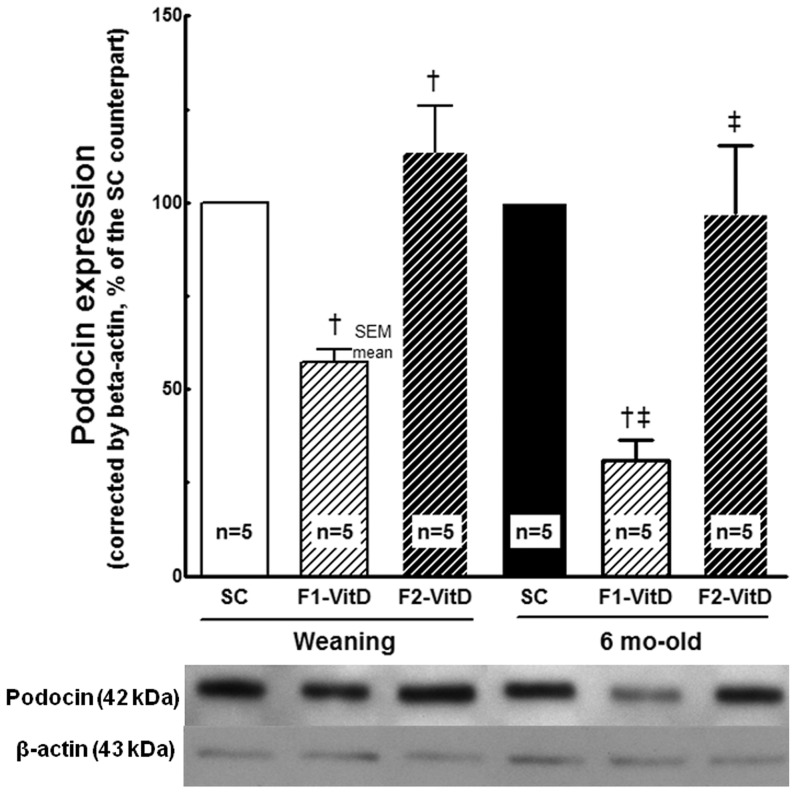
Western blotting analysis of renal tissue at weaning and 6 months of age for podocin expression. The average values were measured and equal protein loading was confirmed by probing blots with beta-actin antibody and is expressed as a percentage of the SC counterpart. Data are reported as means ± SEM; P<0.05, one-way ANOVA and post-hoc of Tukey's test: † compared to SC counterpart; ‡ compared to F1 generation.

F1-VitD- offspring were weaker than the F1-SC offspring. Expression of podocin was 45% weaker at weaning (*P* = 0.001) and 70% weaker at 6 months of age in the VitD- mice than in the SC mice (*P* = 0.0004). However, there was no significant difference in podocin expression between the F2-VitD- and the F2-SC offspring. Therefore, podocin expression was significantly different between the F1 and F2 generations among VitD- offspring at weaning (*P* = 0.02) and at 6 months of age (*P* = 0.004).

#### WT1 expression (Figure 11)

**Figure 11:**
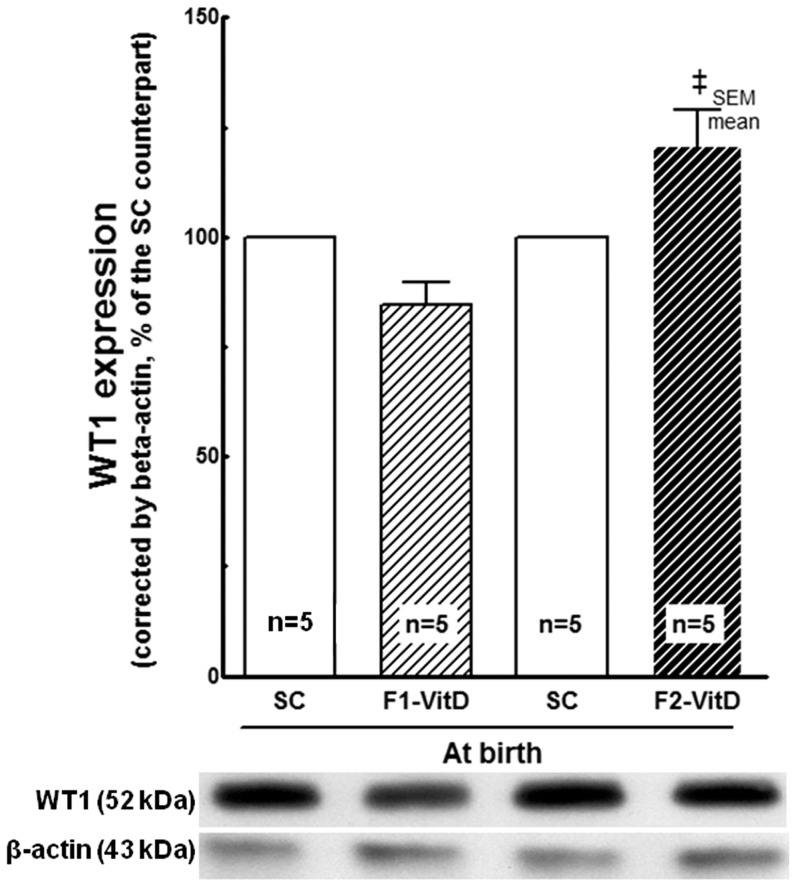
Western blotting analysis of renal tissue at birth for WT1 expression. The average values were measured and equal protein loading was confirmed by probing blots with beta-actin antibody and is expressed into a percentage of the SC counterpart. Data are reported as means ± SEM; P<0.05, one-way ANOVA and post-hoc of Tukey's test: † compared to SC counterpart; ‡ compared to F1 generation.

There was no difference in the expression of WT1 at birth in either the F1 or F2 generations between the SC and VitD- mice (*P*>0.05, two-way ANOVA).

## Discussion

Micronutrient deficiency during development, such as maternal vitamin D deficiency, is a subject of increasing importance because of the nutritional deficiencies caused by excessive consumption of junk food during pregnancy. However, vitamin D insufficiency is still under-recognized and under-treated, despite its prevalence. A recent study of pregnant women in Europe reported that only 12% had an optimal level of vitamin D [20]. In the present study, maternal vitamin D deficiency in mice led to significant changes in kidney development in the offspring, such as retardation of glomerular maturation and an increase in the number of glomeruli. The expression of the RAS and podocin proteins was also altered. Furthermore, not only the F1 generation but also the F2 generation was affected by the gestational vitamin D deficiency of the F0 generation mice.

Vitamin D is normally stored in the liver and adipose tissue, and after six weeks on a restricted diet, the vitamin D levels were extremely reduced without alteration of calcium or phosphate levels in the rodents [21]. Previous studies in rodents have shown that the restriction of vitamin D for a period of six weeks prior to mating assures vitamin D deficiency during pregnancy [13], [22].

Vitamin D deficiency during the third trimester of pregnancy in humans has been associated with cardiovascular disease, diabetes and obesity in offspring [23], which could be linked to the potential role of vitamin D in the regulation of adiposity [24], [25]. Maternal nutrient restriction causes adiposity in offspring [26], and recent data suggest that maternal vitamin D deficiency affects adiposity, although the mechanisms are not yet fully understood [23]. In the present study, adiposity was not the focus. However, there was a significant increase in BM in both generations of VitD- offspring at six months of age, but the BM was higher only in the F2-VitD- group at weaning. In three different moments of post-natal life (birth, 10-days and 3 months of age), BM was not affected by the maternal diet restricted in vitamin D. At weaning and at six months of age, the VitD- offspring were heavier than the SC offspring in both the F1 and F2 generations. However, at three months of age, the VitD- groups in both the F1 and F2 generations showed a tendency toward increased BM, although the difference was not statistically significant. The small sample size of the groups could explain these results.

In rats, it has been observed that maternal vitamin D deficiency leads to a significant increase in the number of glomeruli, which is a unique and important effect of vitamin D restriction during fetal development [13]. To understand this result, it should be emphasized that nephrogenesis in rodents, unlike in humans, continues after delivery for the first 10 days of postnatal life. This period is roughly comparable to the third gestational trimester of humans [27]. Experimental models have demonstrated that blocking the RAS during development causes changes in the glomerular vasculature that affects the normal maturity of the kidney, although the RAS cascade during kidney development is not fully understood [28], [29].

Research suggests that vitamin D is important for differentiation and maturation during cell proliferation [9] and that it down-regulates renin gene expression in the kidneys [13]. However, in the present study, renin expression was significantly higher at six months of age in both the F1 and F2 generations, and AT1r expression was higher in F1-VitD- offspring than in F1-SC offspring.

Kidney organogenesis is begun and maintained as a result of a series of regulatory molecules. WT1 is expressed in the metanephric blastema and probably plays a key role in podocyte differentiation [12], [30]. In the present study, no significant reduction in the expression of WT1 in the kidneys at birth was observed in either the F1 or F2 generation. Although the VitD- offspring in the present study did not show a reduction in WT1 expression, maternal vitamin D deficiency did alter the ratio of immature-to-mature glomeruli at birth by extending the period of glomerular maturation.

Maternal vitamin D deficiency may also have contributed to an increased number of glomeruli, thus producing smaller glomeruli than in animals fed SC. Indeed, the smaller glomeruli could explain the high BP observed among F1-VitD- offspring and the strong tendency toward high BP observed in the F2 generation. Previous research has documented a negative correlation between the number of glomeruli and BP levels in humans [31] and in rodents [27], [32].

This finding is consistent with the high renin and AT1r expressions observed in the present study. Activation of the RAS may play a significant role in the development of mechanisms that lead to kidney damage [33]. After kidney damage occurs, chronic renal disease progresses through a cycle that passes the damage from lost or damaged nephrons to healthy nephrons. Two hypotheses are currently proposed to explain the progression of chronic renal disease. The results of the present study are consistent with the “overload hypothesis”. This hypothesis suggests that structural and functional adaptations are associated with glomerular hypertension, hyperfiltration, and hypertrophy partially compensated for nephron losses [34].

Podocin provides the structural organization of the slit diaphragm and the regulation of filtration function and seems to be involved in the alteration of the glomerular basement membrane [35]. Nephrin is expressed late in the process of podocyte differentiation and is a locus for the formation of the slit diaphragm, foot process maintenance and physical integrity *in vivo*, but it is dispensable for cell survival and has little impact on gene regulation during glomerular development. Nephrin does not affect podocyte apoptosis and gene expression patterns [36].

In the present study, vitamin D restriction during nephrogenesis reduced podocin expression in the F1 generation from weaning until six months of age but not in the F2 generation. Such effects should be evaluated because podocin serves in the structural organization of the slit diaphragm and the regulation of its filtration function [35]. Although the mature animals did not show significant proteinuria, maternal vitamin D deficiency altered podocin expression in the F1 generation from weaning until six months of age, but not in the F2 generation.

The question of how developmental programming passes to subsequent generations deserves further attention. Intense maternal protein restriction, for example, leads to chronic diseases in adult life, affecting both the F1 and F2 generations [37], [38], and reaching even the F3 generation [39]. Part of the explanation seems to be decreased global methylation in these offspring [40].

The present study has certain limitations. The major aim of the study was to investigate glomerular development and renal structure at birth, 10 days and adulthood in mice born to mothers fed a diet restricted in vitamin D. Although, we examined other variables, such as body mass and 24 h urine, the present study was not designed to investigate renal functional and metabolic consequences but rather was designed to answer the question of whether there were structural, glomerular alterations or transgenerational effects. Further studies are necessary to clarify the impact of maternal vitamin D restriction on the renal function of offspring. Furthermore, the literature shows some relationship between vitamin D deficiency and adipose tissue development, and other studies should evaluate such effects, and the transgenerational effects.

In conclusion, the present findings provide insight into the relationship between maternal vitamin D deficiency and the occurrence of a greater number of glomeruli in offspring. Maternal vitamin D deficiency is accompanied by changes in the renal expression of podocin, renin and AT1 receptors, and it delays the maturity of the glomeruli, by extending nephrogenesis. These findings are more pronounced in F1 progeny, but F2 progeny are also affected.
